# Supplementary material for: Improving Confidence in Performing Clinical Procedures Through Peer-Driven Training Sessions for Preclinical Medical Students
Source: MedEdPORTAL. 2025 Aug 19;21:11542. doi: 10.15766/mep_2374-8265.11542 (PMC12361509; doi:10.15766/mep_2374-8265.11542)
Supplement: Supplementary file 1 — Survey.docxI&D Video.mp4Suture Video.mp4Intubation Video.mp4PIV Video.mp4I&D Guide.docxSuture Guide.docxIntubation Guide.docxIV Guide.docxFocus Group Questions.docx [file mep_2374-8265.11542-s001.zip › H. Intubation Guide.docx]

**Endotracheal Intubation**

**Instructions for Facilitator**

This rotation will introduce student-participants to the skill of inserting an endotracheal tube for mechanical ventilation. There will be 4 airway trainers provided in the room. Student-teachers will have a mannequin with a video guided laryngoscope which will allow students to visualize the airway as you demonstrate the procedure. An assistant can be made available for the procedure demonstration and to assist students during the practice time. Students will be broken into groups of two to practice intubation on an airway task trainer with a plastic laryngoscope. Both the instructor and the assistant can then go around the room to assist students as they practice on the mannequins.

**Learning Objectives**

By the end of this rotation, student learners should:

1. Assemble the basic supplies needed to perform an endotracheal intubation.
2. Perform an endotracheal intubation on a simulation mannequin with direct laryngoscopy and video-assisted laryngoscopy.
3. Identify indications for intubation and associated complications such as esophageal intubation.

**Supplies**

| (4) 7.5 Cuffed Endotracheal Tubes | (4) Rigid Stylets |
| --- | --- |
| (4) 10cc Syringes | (4) #3 or 4 Macintosh Laryngoscopes |
| (4) Adult Airway Task Trainers/Mannequins  (4) Bag Valve Masks | (4) Rolls of Tape  (4) Stethoscopes |

**Station Setup**

Below is an example of the station setup that we utilized in our own student led procedure training session. However, moderations can be made as needed based on resources available.


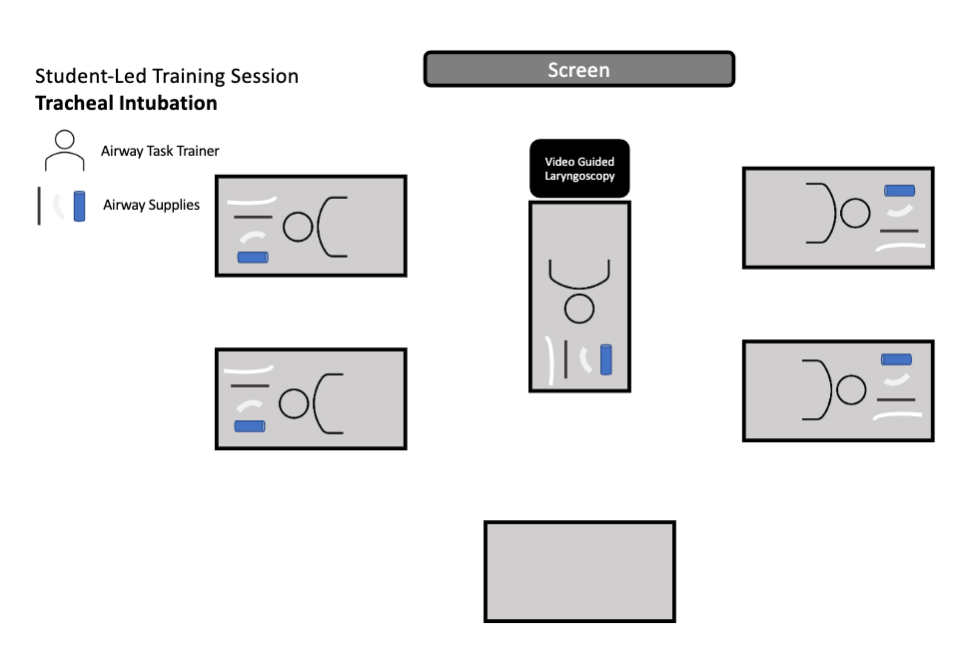


Image 1: Image owned by author.

**Pre-Requisites**

Prior to the workshop learners will be asked to watch a video on endotracheal intubation utilizing a video created by the author (Intubation Video – Appendix D). Alternatively, instructors may choose to provide didactic material utilizing their own slides and/or videos.

**Training Session Outline**

1. **Introduction (5 minutes)**
   1. Instructor should provide brief overview of the indications and contraindications to the procedure. It is important to note that this is not meant to be an extensive course on airway management and the learning objectives for this rotation are focused at introducing students to the physical maneuvers of a direct laryngoscopy endotracheal intubation.
   2. Tools used for intubation
      1. Laryngoscope
         1. Base – provides the power for the light used to visualize airway anatomy as well as serving as the handle of the laryngoscope.
         2. Blade – Used to improve visualization of airway and hold structures in place when inserting ET tube.
      2. Endotracheal (ET) Tube
         1. Tube size is printed on the side of the tube – based on the diameter and measured in millimeters.
         2. Inserted into trachea and used to mechanically ventilate with a BVM or through a ventilator.
         3. Balloon filled with 10cc air syringe used to inflate the pilot balloon.
      3. Stylet
         1. Passed through the ET tube provides sturdier shape by increasing its stiffness and enhancing the control of the tip of the tube. Stylet will be removed after ET tube has been secured in the airway.
      4. Bag Valve Mask
         1. Used to pre-oxygenate patients prior to intubation
            1. Connected to oxygen with 15L flow
         2. Connects to ET tube for manual ventilation
2. **Instructor Demonstration (5 minutes)**
   1. **Intubation with Laryngoscope –**
      1. Ensure that all equipment is functioning properly, and that suction and difficult airway devices are within reach.
         1. Light on laryngoscope
         2. Cuff on ET tube
      2. Place patient in sniffing position, elevate head as needed and preoxygenate.
      3. Hold laryngoscope with left hand. Open the patient’s mouth with right hand (scissor technique) and introduce a laryngoscope into the right side of the patient’s mouth.
      4. Push the tongue to the left side of the mouth, slowly advance the blade and identify the base of the tongue, the epiglottis, and the posterior cartilages.
      5. Visualize the vocal cords and arytenoid cartilages. *Do not take your eyes off the cords once they have been identified. Partner should stand on proceduralist’s right side to hand ET tube upon request and inflate cuff.*
         1. Miller (straight) blade should be placed under epiglottis.
         2. Macintosh (curved) blade placed in the vallecula.  *This is what we are using!*
      6. Lift at a 45-degree angle (direction of the laryngoscope handle).
      7. Using direct visualization, pass the ET tube 3-4 cm beyond the vocal cords.
      8. Remove stylet and inflate balloon.
      9. Connect ET tube to ventilatory equipment, secure ET tube with tape and/or commercial device and confirm proper placement.
         1. Chest rises and falls
         2. Auscultation for breath sounds
         3. End-tidal CO2 detection
         4. CXR in a hospital setting
3. **Student-participant practice time (5 minutes)**
   1. There will be four airway stations in every room so that students can practice in pairs of two, one student will perform the tracheal intubation while the second student will assist in handing the ET tube and inflating the balloon.
   2. Walk around the room during this time to help students as needed.

**Procedure Sources**

1. Driver BE, Reardon RF. Tracheal Intubation. In: Roberts JR, Hedges JR, eds. Roberts and Hedges’ Clinical Procedures in Emergency Medicine and Acute Care. 7th ed. Elsevier; 2019:62-110.e6.
