# Supplementary material for: Improving Confidence in Performing Clinical Procedures Through Peer-Driven Training Sessions for Preclinical Medical Students
Source: MedEdPORTAL. 2025 Aug 19;21:11542. doi: 10.15766/mep_2374-8265.11542 (PMC12361509; doi:10.15766/mep_2374-8265.11542)
Supplement: Supplementary file 1 — Survey.docxI&D Video.mp4Suture Video.mp4Intubation Video.mp4PIV Video.mp4I&D Guide.docxSuture Guide.docxIntubation Guide.docxIV Guide.docxFocus Group Questions.docx [file mep_2374-8265.11542-s001.zip › I. IV Guide.docx]

**Peripheral Venous Access**

**Instructions for Facilitators**

This rotation will introduce student-participants to peripheral venous access or simply put, “how to start an IV.” Each student will have an arm task trainer to follow along as you demonstrate and explain the procedure. IV starter kits will be available for each student in the room. An ultrasound machine will also be available for student teachers to provide a better visual of the procedure during their demonstration.

**Learning Objectives**

By the end of this rotation, student learners should:

1. Assemble the basic supplies needed to gain peripheral venous access.
2. Have increased confidence in their ability to obtain peripheral venous access on a simulation mannequin with proper aseptic technique.

**Supplies**

| (4) Tourniquets | (38) alcohol prep pads | (10) Saline locks |
| --- | --- | --- |
| (10) Tape rolls | (38+) gauze pads | (10) saline flushes |
| (38) Tegaderm | (38+) 22G IV Catheters | (2) Sharp’s containers |
| (5) IV trainer arms  (1) box latex gloves | (5) saline bags | (3) IV poles |

**Station Setup**

Below is an example of the station setup that we utilized in our own student led procedure training session. However, moderations can be made as needed based on resources available.


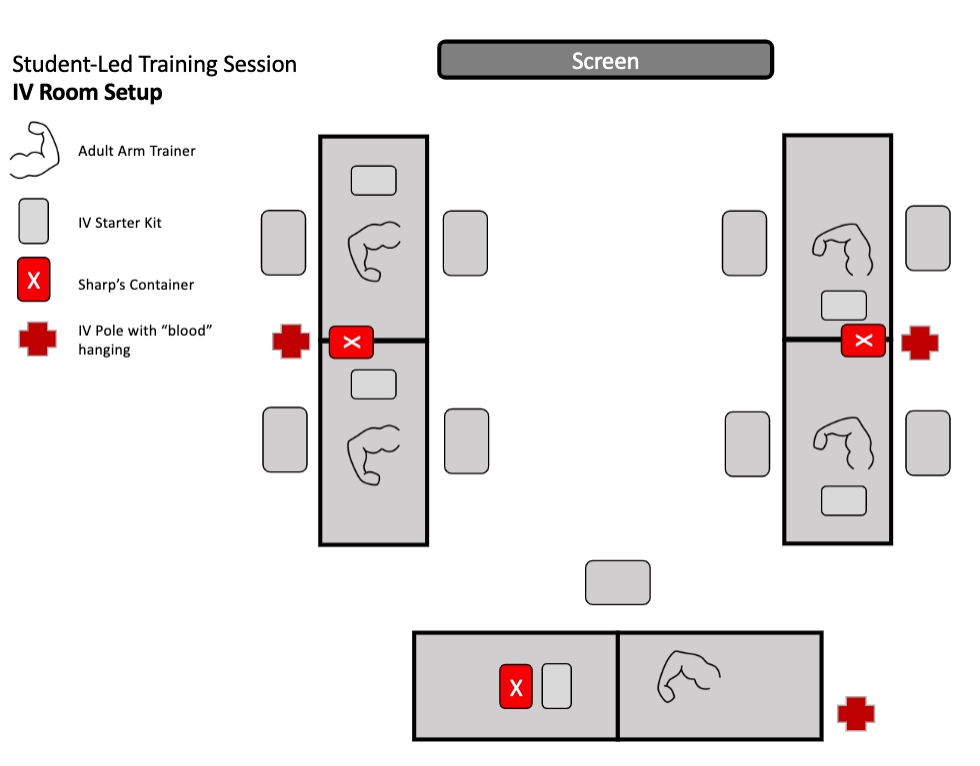


**1**: Author owned image.

**Pre-Requisites**

Prior to the workshop, learners will be asked to watch a video on peripheral venous access utilizing a video created by the author (Peripheral IV Video – Appendix E) . Alternatively, instructors may choose to provide didactic material utilizing their own slides and/or videos.

**Training Session Outline**

1. **Introduction (5 minutes)**
   1. Instructor should introduce the procedure, outline the plan for the rotation, and introduce materials required for the procedure.
2. **Instructor Demonstration (5 minutes)**
   1. **Preparing equipment/supplies before starting an IV**
      1. Prepare IV fluids and tubing
      2. Remove the cap from saline lock
      3. Flush saline lock
   2. **Inspection and Positioning**
      1. Apply tourniquet to arm.
      2. Palpate veins with the index and middle finger of the nondominant hand. The veins are soft, elastic, resilient and pulseless.
      3. Prepare insertion site with an alcohol pad.
   3. **Cannulation**
      1. Insert the IV catheter with the bevel facing upward
         1. Angle IV catheter at 10-30 degrees
      2. Advance the needle until a flash of blood is seen. Once a flash of blood is seen, advance the catheter several millimeters further.
      3. Loosen the stylet and advance the catheter over the needle until flush with the skin.
      4. Press the safety needle button and dispose of needle in sharp’s container.
   4. **Anchoring the Device**
      1. Attach the pre-flushed saline lock.
      2. Cover the insertion site with Tegaderm (or similar dressing) and secure any loose lines with tape.
3. **Student Practice Time (20 minutes)**
   1. With the instructor available to assist, answer questions, and provide feedback, students should be allowed approximately 20 minutes to practice obtaining peripheral venous access on the simulated arm trainers.

**Procedure Sources**

1. Kaplan BL, Liu SW, Zane RD. Peripheral Intravenous Access. In: Roberts JR, Hedges JR, eds. Roberts and Hedges’ Clinical Procedures in Emergency Medicine and Acute Care. 7th ed. Elsevier; 2019:394-404.e2.
