# Supplementary material for: Improving Confidence in Performing Clinical Procedures Through Peer-Driven Training Sessions for Preclinical Medical Students
Source: MedEdPORTAL. 2025 Aug 19;21:11542. doi: 10.15766/mep_2374-8265.11542 (PMC12361509; doi:10.15766/mep_2374-8265.11542)
Supplement: Supplementary file 1 — Survey.docxI&D Video.mp4Suture Video.mp4Intubation Video.mp4PIV Video.mp4I&D Guide.docxSuture Guide.docxIntubation Guide.docxIV Guide.docxFocus Group Questions.docx [file mep_2374-8265.11542-s001.zip › J. Focus Group Questions.docx]

**Pre-Clinical Procedure Skills Session**

**Instructions for Facilitator**

Be sure that your recording device is working properly prior to the beginning of the focus session. When students enter the room, take a moment to explain that you will be asking them a set of open-ended questions which aim to facilitate conversation amongst the members of this focus group. This session should take no longer than 15 minutes. Once everyone is situated, announce that you are going to begin recording and start the audio recording.

**Discussion Questions:** Below is a list of questions which can be used to facilitate conversation amongst the focus group. Facilitators should strive to ask all the questions listed below, but questions can be asked out of order if necessitated by the flow of the conversation. Explain to the group, that this is meant to be a conversation amongst themselves, and they aren’t “responding to the facilitator.” Please keep your personal input to a minimum and allow the student participants to take their conversation where they wish, while kindly redirecting the conversation if they stray too far off topic and moving to the next question once “7 seconds of silence” has passed.

- What went well with the procedure training session?
  - [*begin generally, then prompt about each individual rotation: (1) suturing (2) abscess I&D (3) IV (4) intubation*]
- What could have gone better with the procedure training session?
  - [*begin generally, then prompt about each individual rotation: (1) suturing (2) abscess I&D (3) IV (4) intubation*]
- Which aspects of the session were the best/most informative/most impactful?
- What aspects of the session could have been more informative/more impactful?
- How do you feel that this peer-teaching session compared to prior faculty-led teaching sessions?
- Generally, how effective do you perceive student-teaching to be as a method for teaching clinical procedures to pre-clinical students?
  - Has your opinion changed since the beginning of this session?
- In future sessions, what improvements could be made to make for a better educational experience?
  - Could prompt them to think about things such as:
    - Quality of instruction
    - Supplemental materials
    - Available equipment
    - Time

At the conclusion of the focus group, stop the recording and verbalize that the recording has stopped.
