# Supplementary material for: Improving Confidence in Performing Clinical Procedures Through Peer-Driven Training Sessions for Preclinical Medical Students
Source: MedEdPORTAL. 2025 Aug 19;21:11542. doi: 10.15766/mep_2374-8265.11542 (PMC12361509; doi:10.15766/mep_2374-8265.11542)
Supplement: Supplementary file 1 — Survey.docxI&D Video.mp4Suture Video.mp4Intubation Video.mp4PIV Video.mp4I&D Guide.docxSuture Guide.docxIntubation Guide.docxIV Guide.docxFocus Group Questions.docx [file mep_2374-8265.11542-s001.zip › A. Survey.docx]

**Student Confidence in Performing Procedures**

**Pre/Post-Session Survey**

For the following questions, please indicate your level of confidence in performing the following procedures using a sliding scale from 1-10 where 1 represents the least level of confidence and 10 represents the utmost level of confidence.

My confidence level in my ability to perform basic suturing techniques.

| 1 | 2 | 3 | 4 | 5 | 6 | 7 | 8 | 9 | 10 |
| --- | --- | --- | --- | --- | --- | --- | --- | --- | --- |
| Low Confidence | |  |  |  |  |  |  | High Confidence | |

My confidence level in my ability to perform an incision and drainage on a peripheral skin abscess.

| 1 | 2 | 3 | 4 | 5 | 6 | 7 | 8 | 9 | 10 |
| --- | --- | --- | --- | --- | --- | --- | --- | --- | --- |
| Low Confidence | |  |  |  |  |  |  | High Confidence | |

My confidence level in my ability to perform an endotracheal tube intubation with a laryngoscope.

| 1 | 2 | 3 | 4 | 5 | 6 | 7 | 8 | 9 | 10 |
| --- | --- | --- | --- | --- | --- | --- | --- | --- | --- |
| Low Confidence | |  |  |  |  |  |  | High Confidence | |

My confidence level in my ability to start a peripheral venous catheter (IV).

| 1 | 2 | 3 | 4 | 5 | 6 | 7 | 8 | 9 | 10 |
| --- | --- | --- | --- | --- | --- | --- | --- | --- | --- |
| Low Confidence | |  |  |  |  |  |  | High Confidence | |
