# Supplementary material for: Improving Confidence in Performing Clinical Procedures Through Peer-Driven Training Sessions for Preclinical Medical Students
Source: MedEdPORTAL. 2025 Aug 19;21:11542. doi: 10.15766/mep_2374-8265.11542 (PMC12361509; doi:10.15766/mep_2374-8265.11542)
Supplement: Supplementary file 1 — Survey.docxI&D Video.mp4Suture Video.mp4Intubation Video.mp4PIV Video.mp4I&D Guide.docxSuture Guide.docxIntubation Guide.docxIV Guide.docxFocus Group Questions.docx [file mep_2374-8265.11542-s001.zip › F. I&D Guide.docx]

**Abscess Incision and Drainage**

**Instructions for Facilitator**

This rotation will introduce student-participants to the proper protocol for a peripheral abscess incision and drainage procedure. Each student will have their own simulated abscess created prior to the session so that they can follow along as you demonstrate and explain the procedure.

**Learning Objectives**

By the end of this rotation, student learners should:

1. Have increased confidence in their ability to perform an abscess incision and drainage.
2. Have improved knowledge in the indications for a cutaneous abscess incision and drainage.

**Supplies**

| (36) Simulated abscess* | (9) 25-gauge needle | (36) Gauze strips |
| --- | --- | --- |
| (9) 11# Scalpel | (9) Curved hemostat | (36) Gauze pads |
| (9) 10cc syringe | (9) Local anesthetic vial | (36) Under pads/paper towel |

**Station Setup**

Below is an example of the station setup that we utilized in our own student led procedure training session. However, modifications can be made as needed based on resources available.

**
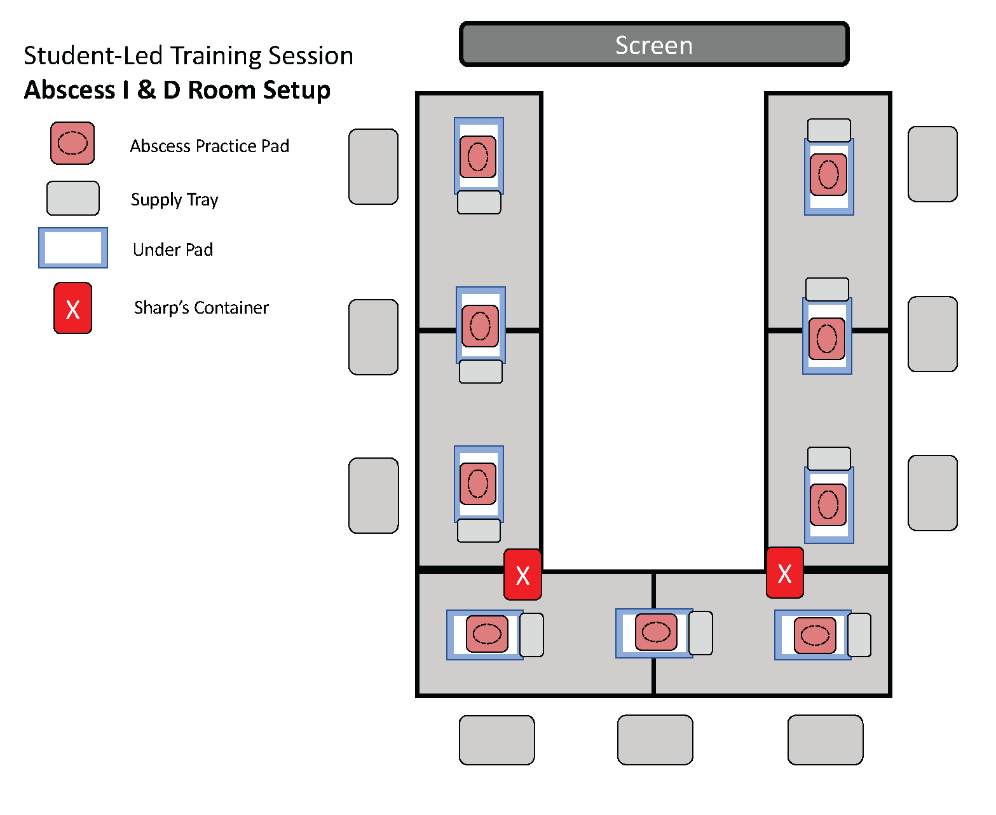
**

**1**: Author owned image.

**Pre-Requisites**

Prior to the workshop learners will be asked to watch a video on abscess incision and drainage utilizing a video created by the author (I&D Video - Appendix B). Alternatively, instructors may choose to provide didactic material utilizing their own slides and/or videos.

**Training Session Outline**

1. **Introduction (5 minutes)**
   1. Instructor should introduce the procedure, outline the plan for the rotation, introduce supplies required and discuss indications for the procedure.
      1. **Indications:** (1) Localized collection of infection/fluid/pus that is tender and is not resolving spontaneously or with conservative measures (2) pain, fever, redness, swelling and loss of function – the cardinal signs of infection are usually present (3) an abscess greater than 0.5 cm (relative) and/or fluctuance.
2. **Instructor Demonstration (10 minutes)**
   1. **Cleaning the field**
      1. Prep the surface with chlorhexidine or povidone-iodine solution. *Will not be done by students, just mention that this needs to be done in clinical practice.* Mention to students that they should wipe inward to outward when applying the antiseptic solution.
   2. **Applying local anesthetic**
      1. **How to draw medication from vial:**
         1. Get the vial ready
            1. If first use from vial, remove cap. Wipe the rubber top clean with an alcohol pad.
         2. Fill the syringe with medicine
            1. Holding the syringe like a pencil, point the needle tip up
            2. Before inserting the needle into the vial, pull the plunger back to the line on the syringe for the dose that will be withdrawn. *You want to inject an equivalent amount of air into the vial as the volume of medication that you will be removing (ex. If you plan on withdrawing 7mL of 1% lidocaine, you should fill the syringe with 7mLs of air and inject that into the vial.*
            3. Insert the needle into the rubber top of the vial
            4. Push the air from your syringe into the vial, preventing a vacuum from forming.
            5. With the needle tip in the rubber top of the vial, turn the vial upside down.
            6. Pull the plunger back to the line on the syringe of the desired volume of medication
      2. **Inject local anesthetic**
         1. First inject a small amount of anesthetic at the vertex of the V (where the dots are in the video). This allows for anesthesia at the two opposite sides of the abscess and minimizes the number of “pokes” that the patient feels if the needle comes out when changing directions, requiring you to reinsert the needle into the skin.
            1. Note: We always want to avoid intravascular injection of anesthetic. Remember to always aspirate/pull back on the syringe plunger before injection. If there is blood return into the syringe, the needle should be repositioned to prevent intravascular injection of the anesthetic.
         2. Next inject anesthetic into superficial tissue along the outside edge of the “V-shape” injection path (remember to try and not remove the needle from the injection site when switching between sides of each “V”) creating a diamond shaped field block around the abscess
         3. Notes:
            1. Additionally, you can inject additional anesthetic into the roof of the abscess in the same location as the planned incision for additional anesthesia (there may be times where the ring/field block isn’t enough).
            2. (1) Abscess is an acidic environment – local anesthetics may lose their effectiveness. Provide adequate amount of anesthetic and allow adequate time for anesthetic to take effect. (2) Avoid injecting into abscess cavity.
   3. **Making a linear incision**
      1. Make a linear incision with an 11 or 15 blade into the abscess. Be sure to provide an incision wide enough to promote adequate drainage.
         1. Use PPE to avoid self-contamination.
         2. Note: The video shown in Appendix B demonstrates the improper use of universal precautions to lessen the risk of a needlestick/sharps where the proceduralist recaps the scalpel in the air. Please note to students that scalpel caps should be laid down and recapped on the field without the other hand actively pushing the blade cover on.
   4. **Techniques for drainage**
      1. Allow purulent fluid to drain from the abscess – use curved hemostats to gently break up loculations and manually express the purulent material from the abscess cavity.
         1. Note: This can be one of the most painful parts for the patient; it is often difficult to get good anesthesia to the deeper tissues and probing around in a forceful manner can be quite painful.
   5. **Irrigation of the abscess**
      1. *Does not need to be demonstrated or performed by students. Mention that this is common practice and how it would be done.*
   6. **Packing the abscess**
      1. Insert packing material into the abscess with hemostats or forceps.
      2. Leave abscess open to allow for adequate drainage post-procedure.
3. **Student Practice Time (15 minutes)**
   1. With the instructor available to assist, answer questions, and provide feedback, students should be allowed approximately 20 minutes to practice performing an abscess incision and drainage on a simulated abscess.
   2. **Alternatively,** students can follow along the instructor step by step for the procedure with an overhead view of the instructor’s field of vision projected on a screen or with slides detailing each step.

*Note: Simulated abscesses were created utilizing materials found on Amazon for less than one dollar per simulated abscess. However, variations of these simulated task trainers can be found online or made at home. See Appendix G: Simulated Abscess Video for a video on how these were made.

**Procedure Sources**

1. Ambrose G, Berlin D. Incision and Drainage. In: Roberts JR, Hedges JR, eds. Roberts and Hedges’ Clinical Procedures in Emergency Medicine and Acute Care. 7th ed. Elsevier; 2019:738-773.e4.
