# Supplementary material for: Improving Confidence in Performing Clinical Procedures Through Peer-Driven Training Sessions for Preclinical Medical Students
Source: MedEdPORTAL. 2025 Aug 19;21:11542. doi: 10.15766/mep_2374-8265.11542 (PMC12361509; doi:10.15766/mep_2374-8265.11542)
Supplement: Supplementary file 1 — Survey.docxI&D Video.mp4Suture Video.mp4Intubation Video.mp4PIV Video.mp4I&D Guide.docxSuture Guide.docxIntubation Guide.docxIV Guide.docxFocus Group Questions.docx [file mep_2374-8265.11542-s001.zip › G. Suture Guide.docx]

**Basic Suturing Techniques**

**Instructions for Facilitator**

During this rotation, students will be instructed on how to perform simple interrupted sutures, an instrument tie, and how to remove simple interrupted sutures. Materials needed for this session can be found below. Instructors will have access to this facilitator’s guide, slides which detail each step of the procedure, and their own set of suturing equipment. The pre-session video covering the techniques included in this training session should be sent to the students prior to the date of the training session.

**Learning Objectives:**

By the end of this rotation, student learners should:

1. List the basic supplies needed to perform basic suturing techniques.
2. Perform simple interrupted suture technique with instrument tie.
3. Perform simple interrupted suture removal.
4. Student learners will display increased comfort with and confidence in their ability to perform basic suturing techniques.

**Supplies**

| (9) Practice Suture Pad | (9) Surgical Scissors | (9) Toothed Forceps |
| --- | --- | --- |
| (9) Needle Driver | (36+) Sutures | (2) Sharp’s Container |

**Station Setup**

Below is an example of the station setup that we utilized in our own student led procedure training session. However, moderations can be made as needed based on resources available.

Image 1: Image author owned. **
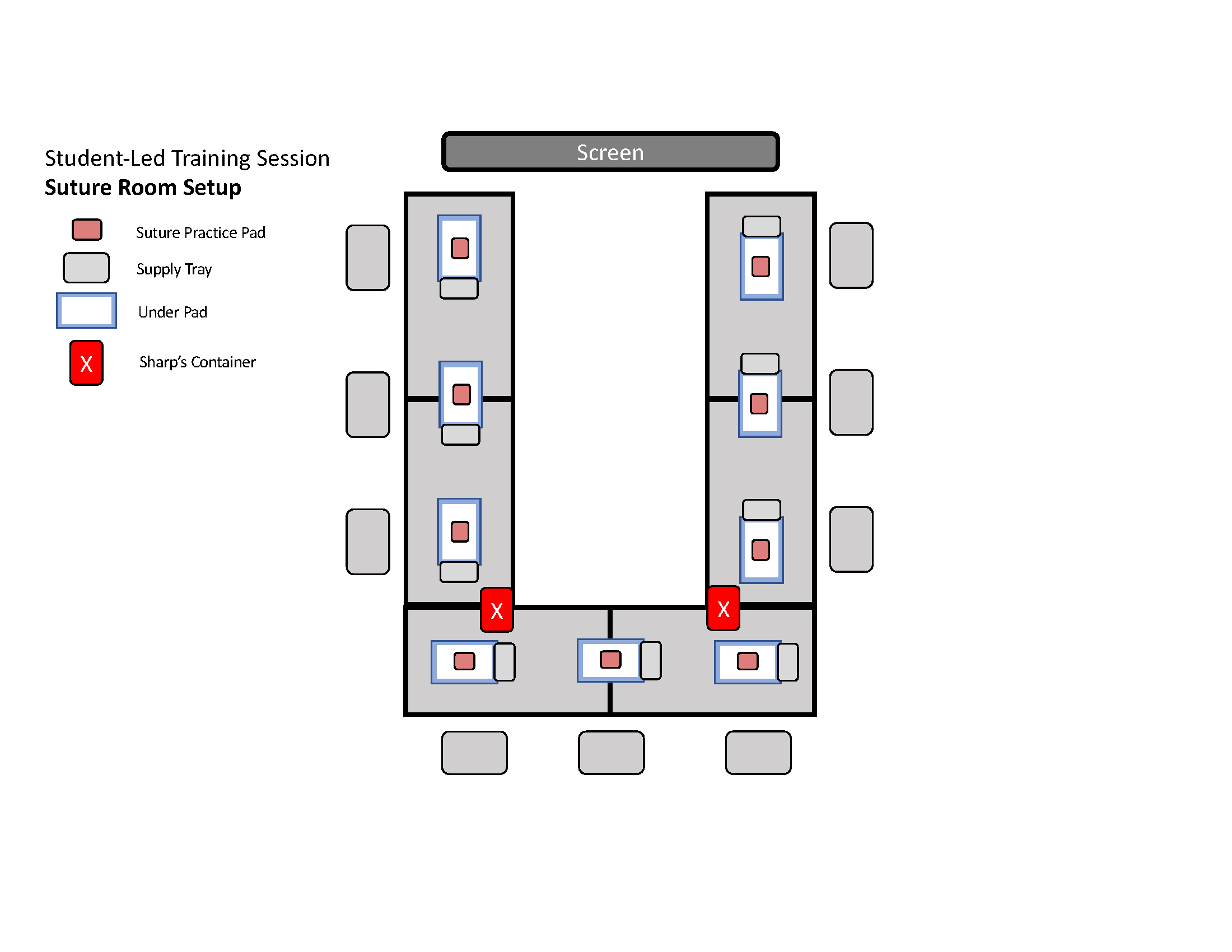
**

**Pre-Requisites**

Prior to the workshop learners will be asked to watch a video on basic suturing techniques utilizing a video created by the author (Suture Video – Appendix C). Alternatively, instructors may choose to provide didactic material utilizing their own slides and/or videos.

**Training Session Outline**

1. **Introduction (5 minutes)]**
   1. Instructor should introduce the procedure, outline the plan for the rotation, introduce supplies required and discuss indications for the procedure. Facilitators should begin the session with a brief background on suturing, introducing the different types of sutures and their indications for use. This is a brief introduction, as the core objective for this rotation is to introduce students to basic suturing technique, while the details of wound care are outside of the spectrum of this training session.
      1. What is ‘suturing?’
      2. Instruments used for suturing
         1. Needle Driver
         2. Forceps (Needle Holder)
         3. Sutures
         4. Scissors
      3. Indications for procedure: Wound or laceration requiring closure that is not amenable to a less invasive method of closure.
2. **Instructor Demonstration (5 minutes)**
   1. **Simple Interrupted Technique**
      1. Hold the needle pointing downward by excessively pronating the wrist so that the needle tip initially moves farther from the laceration as the needle penetrates deeper into the skin. Drive the needle tip downward and away from the cut edge into the subcutaneous layer.
      2. Advance the needle into the laceration. The needle tip is directed toward the opposite side of the same level by rolling the needle holder. The arc of the needle pathway is controlled by retracting the skin edge. This method incorporates more tissue within the stitch in the deeper layers of the wound than at the surface. Alternatively, if a small needle is used or the distance across the wound is great, the needle can be removed from the first side, remounted on the needle holder, and advanced to the opposite side.
      3. Advance the needle upward toward the surface so that it exits at the same distance from the wound edge as on the contralateral side of the wound. Grasp the needle behind the tip and roll it out in the arc of the needle.
      4. Perform an instrument tie as described below.
      5. Close the surface of the wound in segments rather than from one end. Place the first suture in the center of the wound for a straight suture line.
   2. **Instrument Tie**
      1. Place the needle driver parallel to the wound and wrap the suture end twice over the needle driver. This forms the surgeon’s knot, which prevents the first throw from loosening.
      2. Rotate the needle driver 90 degrees and grasp the short suture end on the opposite side of the laceration.
      3. Gently pull the suture ends to the side of the laceration opposite their origin. Tighten only enough to approximate the skin edges; avoid overtightening, which may lead to tissue strangulation.
      4. To begin the second throw, again place the needle driver parallel to the laceration. Wrap the long suture end over and around the needle driver once (only one wrap is used in throws 2 to 4).
      5. Rotate the needle driver 90 degrees and grasp the short suture end on the opposite side of the laceration.
      6. Pull the suture ends to the side of the laceration opposite their origin. On the second and subsequent throws, you can tighten the knot down snugly.
      7. Place an additional two throws (for a total of four). Remember to place the needle driver parallel to the wound and pull the long suture end over the driver; this will ensure that all knots tied are square knots.
   3. **Suture Removal –** *Note: this portion of the session is both to teach the technique, but also to remove the sutures from the previous group so that each participant can have an empty practice board at the start of each rotation*.
      1. Hold scissors in dominant hand and forceps in non-dominant hand.
      2. Grasp the knot of the suture with forceps and gently pull up on the knot while slipping the tip of the scissors under the suture near the skin.
      3. Cut under the knot as close as possible to the skin at the distal end of the knot.
      4. Grasp the knotted end with forceps, and in one continuous action pull the suture out of the tissue and place the removed knot on gauze pad (to be thrown away after all sutures are removed).
3. **Individual Practice Time (20 minutes)**
   1. With the instructor available to assist, answer questions, and provide feedback, students should be allowed approximately 20 minutes to practice performing simple interrupted suturing technique with instrument tie and suture removal.

**Procedure Source**

1. Lammers RL, Scrimshaw LE. Methods of Wound Closure. In: Roberts JR, Hedges JR, eds. Roberts and Hedges’ Clinical Procedures in Emergency Medicine and Acute Care. 7th ed. Elsevier; 2019:655-707.e3.
