# Supplementary material for: Improving Confidence in Performing Clinical Procedures Through Peer-Driven Training Sessions for Preclinical Medical Students
Source: MedEdPORTAL. 2025 Aug 19;21:11542. doi: 10.15766/mep_2374-8265.11542 (PMC12361509; doi:10.15766/mep_2374-8265.11542)
Supplement: Supplementary file 1 — Survey.docxI&D Video.mp4Suture Video.mp4Intubation Video.mp4PIV Video.mp4I&D Guide.docxSuture Guide.docxIntubation Guide.docxIV Guide.docxFocus Group Questions.docx [file mep_2374-8265.11542-s001.zip › _Educational Summary Report_11542.pdf]

MedEdPORTAL® The AAMC Journal of Teaching and Learning Resources

Original Publication Open Access

# Improving Confidence in Performing Clinical Procedures Through Peer-Driven Training Sessions for Preclinical Medical Students

Maxwell B. Bohrer, MD, David L. Rodgers, EdD*

*Corresponding author: rodgerdl@iu.edu

## Abstract

**Introduction:** Preclinical medical students self-report low levels of confidence in performing procedural skills, most often when performing procedures during clinical rotations. Early clinical exposure (ECE) during rotations can increase student confidence in their performance, but resource limitations hinder opportunities for preclinical medical students. A student-led procedure training session (SLPTS) can effectively provide clinical procedure exposure to preclinical medical students outside of the formal medical curriculum, which could increase student confidence prior to the start of clinical rotations. **Methods:** We used Kern's six-step curriculum development approach to create an SLPTS, which included presession videos, procedure background, peer demonstrations, and hands-on practice in suturing technique, abscess incision and drainage, endotracheal intubation, and peripheral vein access. Confidence levels were measured before and after the session using a scale of 1–10 (1 = *low confidence*, 10 = *high confidence*). Mean changes in confidence ($\Delta C$) were analyzed with a paired $t$ test. We conducted focus groups and analyzed common themes using an inductive coding process. **Results:** Among 35 student learners (first year, $n = 17$; second year, $n = 18$; survey respondents, $n = 31$), significant increases in confidence were observed for basic suturing ($\Delta C = 5.16$), abscess incision and drainage ($\Delta C = 5.39$), endotracheal intubation ($\Delta C = 4.55$), and peripheral vein access ($\Delta C = 5.74$; $p < .001$ for all). **Discussion:** The SLPTS effectively provided ECE and increased student confidence in performing clinical procedures outside of the formal curriculum. Collaboration between students and faculty could further integrate such training into the official curriculum.

**Keywords**
Clinical/Procedural Skills Training, Peer Teaching, Emergency Medicine, Focus Groups/Interviews

## Educational Objectives

By the end of this activity, learners will be able to:

1. Demonstrate an increase in self-reported confidence in performing basic suturing techniques, including simple interrupted suture method and surgical knot tying with a needle drive. They will additionally develop the ability to identify appropriate indications and complications of the procedure.
2. Develop improvements in their self-reported confidence in performing an abscess incision and drainage with a single incision method, and in identifying the indications for the procedure.
3. Establish peripheral vein access with proper aseptic technique and have increased self-reported confidence in performing the procedure.
4. Identify indications for endotracheal intubation and recognize complications such as esophageal intubation while obtaining increased self-reported confidence in performing the procedure.

**Citation:**
Bohrer MB, Rodgers DL. Improving confidence in performing clinical procedures through peer-driven training sessions for preclinical medical students. *MedEdPORTAL*. 2025;21:11542.
https://doi.org/10.15766/mep_2374-8265.11542

## Introduction

The AAMC has six foundational competencies for undergraduate medical education, one of which is patient care and procedural skills.[1] However, the delivery of procedure teaching in medical schools across the United States varies widely, and medical students often report low confidence in performing procedural skills during their clinical rotations.[2]

Having early opportunities to receive procedure training during medical school, including hands-on training sessions, is critical to providing students with the confidence to perform procedures during their clinical rotations. Unfortunately,

Copyright © 2025 Bohrer and Rodgers. This is an open-access publication distributed under the terms of the Creative Commons Attribution license. 

these training sessions are often deterred by limited faculty resources. Compared to traditional lectures, an experimental study by Basukala and Chaudhary found that early clinical exposure (ECE) led to better clinical skills and more positive attitudes in preclinical medical students. Faculty members also agreed that ECE enhances students' recall and attitudes, though implementing such courses requires significant faculty resources.[2] Romeo and colleagues also emphasized the significant faculty resource strain required to implement their clinical procedure course for medical students, where attendings and residents served as the instructors.[3]

One approach to overcoming the barriers to implementing clinical procedure training is to involve medical students in designing, facilitating, and delivering clinical procedure sessions.[4] Abay and colleagues noted how medical student peer teaching through simulation was a positive educational experience that decreased faculty burden in an emergency medicine clerkship.[5] There is ample evidence to support the use of peer instructors for medical education. A 2013 randomized controlled study comparing the acquisition of suture skills by novice medical students showed that the student-directed training was similar to the training supervised by a faculty surgeon.[6] Another randomized, single-blinded controlled trial in Malaysia found that peer instruction was not only effective in the delivery of surgical training, but also effective in providing a desired level of procedural competency when compared to faculty instruction.[7] In an investigation of medical student views of peer teaching, Bulte and colleagues found that student learners considered peer teachers well suited for the roles of information providers, role models, and facilitators, but less well equipped to fulfill the role of curriculum planners or resource developers.[8]

This student-led procedure training session (SLPTS) provides a framework by which medical students can take ownership of their medical education through a program that can be replicated with minimal faculty resources, as the program was designed and facilitated by medical students. Our training session shows that with appropriate expert review during the planning phases of curriculum design, medical students can fulfill the roles of curriculum planner and resource developer, creating an effective procedure training session with minimal faculty involvement. This will allow medical students at other institutions to overcome some of the barriers that impede their ability to achieve procedural confidence prior to the start of clinical rotations.

## Methods

A standardized curriculum for the SLPTS was designed by a medical student at Indiana University School of Medicine (Maxwell B. Bohrer) who completed the medical education scholarly concentration program in addition to the core medical curriculum. This scholarly concentration required multiple courses in medical education pedagogy and curriculum design for successful completion. David L. Rodgers, an assistant professor of clinical medicine and doctor of curriculum and instruction, served as mentor for this project. Using Kern's six-step approach to curriculum development,[9] we created a training session with four clinical procedures (Table 1).

### Problem Identification and General Needs Assessment

Preclinical medical students (years 1 and 2 in the traditional medical school curriculum, henceforth called MS1 and MS2, respectively) have little exposure to procedural skills that will become vital during their upcoming clinical training years. Opportunities to learn and practice procedural skills are limited by time demands of the curriculum, availability of faculty, and access to training resources.

The preclinical medical school curriculum typically emphasizes fundamental topics, including anatomy and physiology, pharmacology, pathology, and health system science. Procedural skills are often introduced just prior to starting clinical rotations in transitional courses. While these transitional courses introduce procedural skills, the time between introduction and application is short, leaving little time for building confidence and competence. The ideal approach is to introduce procedural skills early enough to permit time for continued practice to build confidence and competence prior to the start of clinical rotations.[10,11] This approach allows the transitional activities to refresh students' knowledge and skills rather than introduce complex topics and skills immediately prior to clinical rotations. Studies have shown that medical students who are taught procedural skills through adequate simulation-based programs prior to implementing the skills had better performance.[10,11] Additionally, lack of faculty time for instruction may be overcome by other means, including peer teaching.

### Targeted Needs Assessment

Target learners are MS1s and MS2s. This group has requisite knowledge on anatomy and physiology related to clinical skills. Their motivation is high, driven by the anticipation of entering clinical rotations where they will be expected to perform certain skills. Knowledge of the target group indicated the learners would be most receptive to active-learning processes, including hands-on practice with feedback.

Learning new, complex clinical skills may be a stressful activity. Learners will respond better in a low-stress environment geared

Copyright © 2025 Bohrer and Rodgers. This is an open-access publication distributed under the terms of the Creative Commons Attribution license.

**Table 1.** Developing the Student-led Procedure Training Session Through Kern's Six-Step Model of Curriculum Development

| Step | Substep | Content |
|---|---|---|
| 1. Problem identification and general needs assessment | 1a. Health care problem | Preclinical medical students (years 1 and 2 in the traditional medical school curriculum) have very little exposure to procedural skills that will become vital during their upcoming clinical training years (years 3 and 4). Opportunities to learn and practice procedural skills are limited by time demands of the curriculum, availability of faculty, and availability of training resources. |
| | 1b. Current approach | Medical school years 1 and 2 are typically defined as preclinical years involving required fundamental topics such as anatomy and physiology, organ systems, and, in some schools, health system science. Procedural skills are often not introduced in courses and workshops until just prior to the start of clinical rotations, aiming to bridge the transition from preclinical to clinical activities. While these transitional courses introduce the skills, the time between introduction and application is short, leaving very little time for building confidence and competence. |
| | 1c. Ideal approach | The ideal approach is to introduce procedural skills early enough to permit time for continued practice to build confidence and competence prior to the start of clinical rotations. This approach allows the transitional activities to refresh knowledge and skills rather than introduce complex topics and skills immediately prior to clinical rotations. Studies have shown that medical students receiving adequate simulation-based learning for procedural skills prior to implementing the skills had better performance. Additionally, lack of faculty time for instruction may be overcome by other means, including peer teaching. |
| 2. Targeted needs assessment | 2a. Learners | Target learners are preclinical medical students (years 1 and 2). This group has requisite knowledge on anatomy and physiology related to clinical skills. The group is highly motivated due to knowing they will soon be in clinical rotations and be expected to perform certain skills. Knowledge of the target group indicated the learners would be most receptive to active-learning processes, including hands-on practice with feedback. |
| | 2b. Learning environment | Learning new, complex clinical skills may be a stressful activity. Learners will respond better in a low-stress environment geared to formative development of procedural skills with peer teachers compared to the stress of performing in front of faculty. A safe learning environment will be emphasized where learners are free to experiment, make mistakes, and learn skills utilizing simulation. |
| 3. Goals and objectives | 3a. Broad goals | The goal of this program is to introduce early clinical exposure for procedural skills well in advance of clinical rotations, with a peer-to-peer teaching model to allow medical students the opportunity to practice skills in a low-stress environment and build confidence and competence in performing those skills. Early introduction will allow medical students opportunities to evaluate their own performance, recognize areas for improvement, and conduct continued practice prior to the start of clinical rotations. Use of well-prepared peer instructors will reduce dependance on faculty and contribute to a low-stress learning environment. |
| | 3b. Specific measurable objectives | Increase self-reported confidence in performing the following procedures: (1) basic suturing techniques; (2) abscess incision and drainage; (3) peripheral vein access; and (4) endotracheal intubation. See Appendices B–E for further procedure details. |
| 4. Educational strategies | 4a. Content | Content validity for clinical skills was established using resources commonly used in medical student and resident education and training. Clinical content was reviewed with an expert faculty member in emergency medicine who had also completed a simulation fellowship program.<br>Content validity for educational processes was reviewed by an expert educator who has a terminal degree in curriculum and instruction. Specific educational practices were developed from a review of the literature. A key point in the educational methodology was well-delivered peer-to-peer feedback using the Pendleton feedback model. |
| | 4b. Method | Procedural skills require a mix of all three of Bloom's learning domains: cognitive, psychomotor, and affective. The best educational strategy involves: a workshop format that combines preworkshop cognitive knowledge using printed materials, online resources, and video demonstrations; workshop activities that allow practice of psychomotor skills with corrective feedback; and opportunities to build positive affect as demonstrated through increased confidence in performing the procedures.<br>The workshop was designed with respect to the learners' existing knowledge and skill sets and allowed integration of these preexisting sets with the new content being introduced, aligning with the concepts of a constructivist leaning philosophy.<br>As many of the objectives emphasized performance using action verbs (e.g., clean, insert, make), hands-on activities would be required to allow learners to demonstrate psychomotor skills. Creating congruence between objectives and methods is essential. |
| 5. Implementation | 5a. Obtaining support | As the learners are a highly motivated group, anxious to be better prepared for clinical rotations, obtaining learner support faced few hurdles. In total, there were 72 potential subjects representing preclinical medical students on the campus. Because the workshop model emphasized peer-to-peer teaching, the need for many faculty to teach was not present. Only one content expert faculty and one educational expert faculty were required. Both faculty members were readily available and eager to participate. |
| | 5b. Securing resources | The campus has a large simulation center well-equipped with all resources, including mannequins, supplies, and equipment, to facilitate the simulation-based learning workshops. Access to relevant literature is available through the medical library. While video lessons of the specific skills are available online, it was decided to produce specific instructional videos for the program to ensure availability, align video demonstration with the procedural instructions, and reduce risk for copyright infringements. |
| | 5c. Addressing barriers | The primary barrier is aligning time availability for the workshop with learner schedules. Additionally, as a nine-campus medical school, the workshop could not be a required part of the overall medical school curriculum since it was campus specific. Therefore, the recruitment information specifically stated this was an optional educational opportunity and would be conducted in the evening after the normal curriculum had concluded.<br>As a peer teaching exercise, peer instructor content knowledge was required. This barrier was addressed with development of curriculum guides for each station that detailed station-specific instructions with station activity outline, station-specific educational objectives, supplies and equipment, station setup, and information resources. Additionally, each peer instructor was required to attend a simulation-based learning session with the content and education experts to confirm knowledge and skills with each station topic. |

(*Table continues*)

Copyright © 2025 Bohrer and Rodgers. This is an open-access publication distributed under the terms of the Creative Commons Attribution license.

**Table 1.** (*continued*)

| Step | Substep | Content |
|---|---|---|
| | 5d. Introducing the curriculum | The course leader assembled all materials, supplies, and equipment; tested content delivery outlines; and constructed peer instructor documents and resources. Adequate testing was conducted to ensure stations were fully functional and met the content and educational requirements outlined through the objectives.<br>Once testing was complete, the course was prepared for full implementation with the peer instructor training and the peer-to-peer workshop. |
| | 5e. Administering the curriculum | The course was conducted over 2 hours in the Interprofessional Simulation Center. |
| 6. Evaluation and feedback | 6a. Individual learners | The goal of the course was to increase medical student confidence in the performance of select clinical skills. The goal did not include demonstrating competence. To measure confidence changes, pre/postintervention surveys will be obtained using a secure data collection tool. Each survey consisted of questions regarding students' level of confidence in performing the procedures on a scale of 1–10 (1 = *low confidence*, 10 = *high confidence*). Using a paired *t* test with a 95% confidence interval, the change in confidence from preintervention to postintervention for all four skills was measured using Microsoft Excel data analysis tools. |
| | 6b. Program | A qualitative evaluation of the learning event will be obtained through focus group interviews. These interviews will be coded to reflect themes using an inductive coding process. Semistructured interviews for focus groups will include information on evaluating program success in achieving objectives. |

to formative development of procedural skills facilitated by peer teachers, compared to the higher stress of performing in front of faculty.[12] We emphasized a safe learning environment where learners are free to experiment, make mistakes, and learn skills utilizing simulation.[13] Thus, the goal of this peer-to-peer teaching program is to introduce ECE to preclinical medical students to learn procedural skills well in advance of clinical rotations, which will allow the students to practice these skills in a low-stress environment and could build their confidence and competence in performing clinical procedures. Use of well-prepared peer instructors will reduce dependence on faculty and contribute to a low-stress learning environment.

### Educational Strategies

Content validity for clinical skills was established using resources commonly implemented in medical student and resident education and training. Clinical content was reviewed with an expert faculty member in emergency medicine who had also completed a simulation fellowship program. Content validity for educational processes was reviewed by an expert educator who has a terminal degree in curriculum and instruction. Specific educational practices were developed from a review of the literature. A key point in the educational methodology was well-delivered peer-to-peer feedback using the Pendleton feedback model.[14,15] This model is a learner-centered approach to giving feedback in which the learner states what went well, followed by identifying what could have been improved. The teacher then reinforces what went well and provides suggestions for improvement.

### Implementation

The SLPTS was implemented electively, outside of the formal medical curriculum, in an evening session separate from the students' required curricular activities. Preclinical medical students at a regional medical campus were eligible for enrollment, and all eligible students received an email requesting participation in the program. We recruited students to serve in the role of either peer instructor ($n = 7$) or learner ($n = 37$). After providing consent for enrollment, students completed a presession survey, created by the lead author (Maxwell B. Bohrer), containing questions about their confidence in performing procedures (Appendix A), similar to the methods used by Klumpp and colleagues.[16] This survey was reviewed by David L. Rodgers, as well the emergency medicine physician who was also present for the peer instructor training session, as described below.

The peer instructor group attended a training session 1 week prior to implementing the SLPTS. They received training on how to perform peripheral vein intravenous (IV) access, endotracheal intubation, abscess incision and drainage, and basic suturing procedures (Appendices B–E). These procedures were selected from a list of procedural opportunities, created by the Society of Academic Emergency Medicine, that are commonly encountered by medical students in the emergency department.[17] An emergency medicine physician, who had completed a medical simulation fellowship and serves as the campus simulation champion, was present during the training. To assist with the training session and ensure that proper technique was being instructed prior to the SLPTS, the peer instructors were provided with facilitator guides for each procedure (Appendices F–I), designed by Maxwell B. Bohrer. The peer instructors were provided with these facilitator guides prior to both training sessions. This training session additionally served as our pilot for the SLPTS, in which revisions could be made prior to implementing the SLPTS. Peer instructors additionally received

Copyright © 2025 Bohrer and Rodgers. This is an open-access publication distributed under the terms of the Creative Commons Attribution license. 

orientation from an expert simulation educator (David L. Rodgers) on providing feedback in the context of simulation-based skills learning activities. No prerequisite knowledge was necessary prior to the training session. All peer instructors were in their first or second year of medical school.

Students in the peer instructor group then facilitated the SLPTS, where they taught their peers the four procedures presented during the initial training session. Each rotation consisted of a brief introduction to the procedure, including indications and contraindications to each procedure, a facilitator demonstration, and student practice time. Author-owned presession videos were made available through digital links that were emailed to the peer instructors and learners prior to the event (Appendices B–E). Each rotation was 30 minutes. The total time for the training session was 2 hours. Each station consisted of one or two instructors and eight learners. Each station was held in a separate room within our institution's simulation center.

The four simulation-based skills stations were:

*Station 1: abscess incision and drainage* (Appendix F): Following a brief introduction, peer instructors demonstrated how to perform a local anesthetic field block, followed by an incision and drainage utilizing a simulated abscess. Learners then practiced the procedure as the instructor assisted, answered questions, and provided feedback.

*Station 2: simple interrupted suturing technique* (Appendix G): Following a brief introduction to the procedure, including a summary of baseline suture terminology, peer instructors demonstrated how to perform the simple interrupted suturing technique, carry out the instrument tie, and remove sutures. Peer instructors utilized an overhead camera connected to a screen in the room to ensure optimal viewing. Learners then practiced the procedure as the peer instructor assisted, answered questions, and provided feedback.

*Station 3: endotracheal Intubation* (Appendix H): Learners were provided a brief introduction to endotracheal intubation, emphasizing that the purpose of the training session was to introduce the physical maneuvers of the procedure. Details regarding intubation medication, specific indications and contraindications, as well as back-up airway options were beyond the scope of the station. Learners observed the peer instructors as they demonstrated the procedure, utilizing a self-constructed video laryngoscope for the simulation,[18] which was connected to a screen in the room, allowing learners to visualize the anatomy of the mannequin as the peer instructor demonstrated the skills. Learners then practiced the procedure via direct laryngoscopy as the peer instructor assisted, answered questions, and provided feedback.

*Station 4: peripheral vein IV access* (Appendix I): Learners observed the instructor perform the peripheral vein IV access procedure on a task trainer arm. Learners then broke into groups and practiced the procedure as the peer instructor assisted, answered questions, and provided feedback.

Each guide included facilitator instructions with station activity outline, station-specific educational objectives, supplies and equipment, station setup, and information resources.

### Evaluation and Feedback

We evaluated the session using a one-group presession/postsession quantitative survey (Appendix A) and by analyzing feedback from the postintervention focus groups (Appendix J). The Indiana University Institutional Review Board approved the project as exempt from further review (protocol 17409). All students consented to their participation.

*Quantitative data analysis:* Learner survey responses regarding their confidence levels in performing each of the procedures, obtained prior to and after participation in the student-led training session, were recorded using a secure data collection tool (Appendix A). Each survey consisted of questions regarding students' level of confidence in performing the procedures on a scale of 1–10 (1 = *low confidence*, 10 = *high confidence*). Using a paired *t* test with a 95% confidence interval (95% CI), the mean change in confidence levels for all four skills from presession to postsession was measured using Microsoft Excel data analysis tools.

*Qualitative data analysis:* At the conclusion of the SLPTS, learners were divided into seven groups of five participants per group. In each group, learners participated in 15-minute focus group interviews, which were recorded, manually transcribed, and analyzed for common themes identified throughout the discussions using an inductive coding process. Peers of the student learners (first- and second-year medical students) moderated the focus groups using a set of guiding questions to initiate conversation. Maxwell B. Bohrer created the focus group guide, which was reviewed by David L. Rodgers as well the seven focus group moderators prior to implementation in the SLPTS. Maxwell B. Bohrer reviewed and categorized the data, ensuring a comprehensive interpretation of the findings.

Copyright © 2025 Bohrer and Rodgers. This is an open-access publication distributed under the terms of the Creative Commons Attribution license.

## Results

Quantitative Data Analysis

Forty-four learners participated in the SLPTS, of whom seven served in the peer instructor role and 37 were part of the learner group (MS1, $n = 18$; MS2, $n = 19$). The group consisted of 21 female participants and 16 male participants. Of the 37 students who enrolled in the educational program, two did not complete the presession survey, and two who completed the presession survey were unable to attend the training session and thus did not complete the postsession survey. Additionally, two learners who completed the presession survey and attended the session did not complete the postsession survey. Thus, a total of 31 learners completed both the presession and postsession surveys regarding confidence levels and were included in the quantitative data analysis. Thirty-five SLPTS student learners attended the postsession focus groups.

The 31 student learners included in the quantitative data analysis showed significant increases in confidence after participating in the SLPTS. Statistically significant improvements in mean confidence levels in performing all four procedures ($p < .001$ for all) were noted by the survey respondents (Table 2). The largest improvement in confidence was seen in the peripheral vein IV access station, where we observed a mean difference between presession and postsession confidence scores of 5.74 (95% CI 4.84 to 6.64). The smallest improvement in confidence was seen in the intubation station, with a mean difference in confidence scores of 4.55 (95% CI 3.62 to 5.48).

Qualitative Data Analysis

We identified three themes from the SLPTS focus group discussions: (1) high level of organization; (2) creation of a comfortable learning environment; and (3) effective peer teaching. For each theme we identified additional subthemes (Table 3).

*Theme 1: high level of organization:* Learners appreciated the aspects of the SLPTS that highlighted the attention to detail paid to its design and implementation. Learner feedback emphasized the efforts made to incorporate a variety of learning styles throughout the curriculum design process. The learners indicated that the program felt highly organized, given the attention to detail in creating presession videos, visual aids, as well as peer-led demonstrations for each procedure. One student mentioned, "having all different types of learners, where some people learn by seeing it, hearing it, or doing it, the resources available accommodated all of those needs." Most students expressed that the time spent at each station was adequate. One student commented, "compared to past sessions that we have had, in effectively the same amount of time, we got to do more things, more repetitions, more variety in what we were doing." However, a few students mentioned that they might have benefitted from more time at each station. Having adequate supplies was critical; as one student stated, "having enough supplies was a huge thing, it was just very well thought out, there were plenty of supplies for everyone." The program received praise regarding the content, with one student commenting, "I have gotten more hours out of these 2 hours than I have in my entire clinical contact hours."

*Theme 2: creation of an optimal learning environment:* As the most prevalent theme overall, learners indicated that an optimal learning environment was created during the training session. Student feedback demonstrated that the SLPTS was a question-friendly atmosphere with reduced intimidation compared to faculty-led training sessions. The atmosphere created in the SLPTS promoted question-asking by student learners, with one student stating, "I felt more comfortable asking questions that I would have otherwise been hesitant to ask." Learners found the safe learning environment to be ideal for an introductory procedure training session, with one student mentioning that "when there is a faculty member teaching, it is great because they have a vast amount of knowledge, but when there is a peer teaching, I can be comfortable." Ultimately, learners agreed that this experience was one that should be included in their formal medical curriculum.

*Theme 3: effective peer teaching:* Overall, learners reported that peer teaching was an effective method for receiving an introduction to the clinical procedures taught in this session.

**Table 2.** Change in Confidence of Learners in Performing Four Procedures ($N = 31$)

| Procedure | *M* Baseline Score[a] | *M* Postsession Score[a] | *M* Difference (95% CI) | *p* |
|---|---|---|---|---|
| Abscess I&D | 2.13 | 7.52 | 5.39 (4.57 to 6.21) | <.001 |
| Suturing | 2.06 | 7.23 | 5.16 (4.31 to 6.01) | <.001 |
| Intubation | 2.00 | 6.55 | 4.55 (3.62 to 5.48) | <.001 |
| Peripheral IV Access | 2.19 | 7.94 | 5.74 (4.84 to 6.64) | <.001 |

Abbreviations: I&D, incision and drainage; IV, intravenous.
[a]Rated on a 10-point scale (1 = *low confidence*, 10 = *high confidence*).

Copyright © 2025 Bohrer and Rodgers. This is an open-access publication distributed under the terms of the Creative Commons Attribution license.

**Table 3.** Qualitative Data Analysis (*N* = 37) From Focus Group Session

| Major Theme | Subtheme | Quotes |
|---|---|---|
| 1. High level of organization | 1.1 Resources | "This was just way more organized than what we have seen previously. I think you guys did a really good job of meeting us where we are at and used different methods of showing us how to do things."<br>"Having all different types of learners, where some people learn by seeing it, hearing it, or doing it, the resources available accommodated all of those needs." |
| | 1.2 Structure | "Compared to past sessions that we have had, in effectively the same amount of time, we got to do more things, more reps, more variety in what we were doing."<br>"I thought the timing was really good too, you didn't spend so much time in one place, it was enough time to do something, but not so much that you were sitting there waiting around for the next one to start."<br>"Very organized, I think that the [program director] did a great job planning out how the day went. There wasn't chaos, like some of our other events [in formal curriculum] have gone." |
| | 1.3 Supplies | "I don't know if I was surprised or impressed, or both, by the equipment and the setup—very well done."<br>"I think having enough supplies was a huge thing, it was just very well thought out, there were plenty of supplies for everyone." |
| | 1.4 Content | "I have gotten more hours out of these 2 hours than I have in my entire clinical contact hours."<br>"This whole session has been probably my best learning experience in medical school." |
| 2. Optimal learning environment | 2.1 Question friendly atmosphere | "I thought it was nice because I felt more comfortable asking questions that I would have otherwise been hesitant to ask."<br>"I think I am also much more comfortable asking questions and asking for help when I need it when it is a peer [teacher] because we know what style of learning works for each other." |
| | 2.2 Reduced intimidation | "When there is a faculty member teaching, it is great because they have a vast amount of knowledge, but when there is a peer teaching, I can be comfortable."<br>"Having a set of students who have already gone through it made it a lot more relaxing, more friendly, a lot less stressed, nobody was getting 'pimped,' it was a nice atmosphere. |
| | 2.3 Call for inclusion in formal curriculum | "I think too, something should be said about getting this clinical experience earlier on in our-pre-clinical time. Even in the first year, looking at the first years that are here, they're enjoying it. They are enthused to be here, and I think it is so easy to say, 'yeah you are in your pre-clinical years, and you are supposed to be in the books,' but we are here to help patients and a lot of that is hands on. So, I think that directing that focus in other methods and getting re-energized about medicine is great." |
| 3. Effective peer teaching | 3.1 Peer-teacher preparation | "I feel like for the procedures we did, the training you [student instructors] did beforehand [faculty-led training session] was enough to teach us the procedures."<br>"I thought there was great preparation, all of the instructors knew what they were doing." |
| | 3.2 Proximity to learners | "I feel like having someone closer to our age, you know MS1s/MS2s, they understand kind of how you're thinking or the anxiety that you have when learning something new because they just went through that recently, so I feel that they're better equipped to teach us." |
| | 3.3 Change in perspective of peer teaching | "This further strengthened my opinion that peer teaching is a good way of learning, you have people who are, I wouldn't say experts, but they have a lot of knowledge in a certain area and are willing to spread that knowledge."<br>"If anything, I thought it was going to be very effective, but this showed me that peer-teaching can be more effective than I thought it was going to be."<br>"I didn't think it was going to be very useful, but I was proven wrong." |

Abbreviations: MS1, first-year medical student; MS2, second-year medical student.

Moreover, learners were happy with the level of preparation made by their peers but found certain instructors to be more prepared than others. One student commented, "I feel like having someone closer to our age, you know MS1s/MS2s, they understand kind of how you're thinking or the anxiety that you have when learning something new because they just went through that recently, so I feel that they're better equipped to teach us," thus showing that intellectual proximity of peer instructors to the learners was beneficial to the educational experience. Additionally, the session created a positive change in many of the learners' perspectives of peer teaching. One student stated, "I didn't think it was going to be very useful, but I was proven wrong."

## Discussion

In this educational intervention, we have described a peer-to-peer teaching SLPTS that may have distinct advantages over programs designed by faculty. Students showed a significant increase in their confidence in performing all four procedures following participation in this SLPTS, consistent with previously described utilization of peer- and near-peer teaching within the medical education literature. Similar increases in confidence among MS1s and MS2s have also been demonstrated in faculty-led procedure training sessions.[16] Analysis of themes from the focus group discussions showed that the procedure training session had an effective design and adequate resources.

Having a high level of organization was of utmost importance as we prepared for the implementation of the SLPTS. Previous literature has reported that programs designed and developed by medical students have a high level of efficacy.[19,20] However, no previous studies have evaluated the efficacy of a procedure training program that is not only facilitated by medical students as peer instructors but also designed in its entirety by medical

Copyright © 2025 Bohrer and Rodgers. This is an open-access publication distributed under the terms of the Creative Commons Attribution license.

students. The study from Bulte and colleagues on student views of peer teaching showed that peer teachers were equipped to facilitate training sessions, providing their peers with information, but did not have the skill set to plan and develop the resources needed for such a training session.[8] With this in mind, a significant amount of time was devoted to the preparation of facilitator guides, original videos utilizing the same materials that would be used during the SLPTS, as well as in-person demonstrations. Contrary to findings from the Bulte and colleagues study, the qualitative results obtained from focus groups showed that the resources and overall organization of the SLPTS were consistently appreciated as one of the program's greatest attributes.

Similar to previous studies comparing peer teaching to faculty teaching,[21] the creation of an optimal learning environment was an aspect of our curriculum that is somewhat unique to student-led training sessions. Learners can often feel stressed during training sessions led by faculty members. Although peer instructors do not have the level of knowledge held by faculty members, learners found the safe learning environment to be ideal for an introductory-level procedure training session.

Consistent with the findings of research by Lockspeiser and colleagues,[22] our learners indicated that the session was successful due to the intellectual proximity of peer instructors to the learners, a distinct advantage over faculty-led training sessions. While some students doubted that the training session would be beneficial to their procedure skills, there was overwhelmingly positive feedback regarding the value that this session provided to their educational experience. The positive change in perspective on the use of peer instructors outlined from students within the focus groups is a key component of adult learning theory, specifically transformative learning theory as described by Mezirow.[23] Learner beliefs that led to doubts on the peer instructors' capabilities and course outcomes were challenged as the experience unfolded. This exemplifies Mezirow's concept of a disorienting dilemma, as prior beliefs evolved based on the experience until a new belief was adopted.

The reasoning behind this feedback may be attributable to several factors. First, as described earlier, the intellectual proximity of a medical student peer instructor to their learner audience may provide the opportunity to recognize specific needs and desires within their curriculum. Faculty-developed programs may be reused year-to-year with minimal changes that may not account for the evolving medical student demographics and state of medical education. Additionally, faculty members may rely on end-of-course evaluations as their primary method of feedback from students, which may not be representative of the feedback that would be given by students immediately after a specific educational session. Using this model, medical students at other institutions can implement their own SLPTS, allowing them to increase their procedural exposure and take ownership over their medical education.

This educational training program is limited in its ability to provide objective evidence for improvement in students' skills to perform clinical procedures, because there was no formal assessment performed before and after the training session. However, previous studies have shown that a student's level of confidence in performing a procedure is associated with their actual ability to perform the procedure following training.[24] Thus, change in confidence may be an appropriate surrogate for procedure performance to evaluate the efficacy of student instruction. An additional concern is that peer instructors may teach improper procedure techniques. This concern was mitigated by requiring all peer instructors to participate in a training session prior to the SLPTS, in which an emergency medicine faculty member was present to ensure proper technique was taught. This faculty member additionally reviewed the facilitator guides and provided suggestions for revision prior to implementing the SLPTS.

Future research should be conducted to measure skill acquisition using a standardized assessment tool in a head-to-head study comparing peer instructors with faculty instructors. A standardized method of assessing a student instructor's procedural skills could also be useful, ensuring that all instructors begin with a desired level of competence prior to the SLPTS. Comparing student learners in their clinical years who participated in the SLPTS to those students who did not participate, in order to evaluate long-term changes in confidence and objective procedural skill retention, could provide further support for this method of educational delivery. Following student learners throughout their medical career and measuring the impact of this training session on specialty selection is another area of interest that could be evaluated.

## Appendices

A. Survey.docx

B. I&D Video.mp4

C. Suture Video.mp4

Copyright © 2025 Bohrer and Rodgers. This is an open-access publication distributed under the terms of the Creative Commons Attribution license.

D. Intubation Video.mp4

E. PIV Video.mp4

F. I&D Guide.docx

G. Suture Guide.docx

H. Intubation Guide.docx

I. IV Guide.docx

J. Focus Group Questions.docx

*All appendices are peer reviewed as integral parts of the Original Publication.*

**Maxwell B. Bohrer, MD:** Resident Physician, Department of Emergency Medicine, Indiana University School of Medicine; ORCID: https://orcid.org/0000-0003-4364-5952

**David L. Rodgers, EdD:** Assistant Professor of Clinical Medicine, Department of Medicine, Indiana University School of Medicine; Director, Interprofessional Simulation Center, Indiana University Bloomington; ORCID: https://orcid.org/0000-0003-1559-9987

### Acknowledgments
Special thank you to Matthew Needler and Paul VandeKoppel, MD, for their help with this project and support that they give to the medical students at Indiana University School of Medicine.

### Disclosures
None to report.

### Funding/Support
None to report.

### Prior Presentations
Bohrer M, Rodgers D. Evaluating the efficacy of a procedure training session for pre-clinical medical students designed and facilitated by medical students. Poster presented at: IUSM Medical Education Day Conference; April 28, 2023; Indianapolis, IN.

Bohrer M, Rodgers D. Developing future educators through a peer-led procedure training session. Poster presented at: IUSM Medical Education Day Conference; April 28, 2023; Indianapolis, IN.

### Ethical Approval
Indiana University Institutional Review Board reviewed this project.

## References

1. *Foundational Competencies for Undergraduate Medical Education*. AAMC, AACOM, ACGME; 2024. Accessed July 8, 2025. https://image.email.aamc.org/lib/fe8e13727c63047f73/m/1/3d55a3aa-b3ba-4c14-a447-8c60e27a6f05.pdf
2. Basukala A, Chaudhary K. Early clinical exposure in preclinical years of medical school. *JNMA J Nepal Med Assoc*. 2021;59(242):1072-1074. https://doi.org/10.31729/jnma.5341
3. Romeo R, Blasiole B, Chalifoux T, et al. A clinical procedures course for medical students. *MedEdPORTAL*. 2016;12:10524. https://doi.org/10.15766/mep_2374-8265.10524
4. Bennett SR, Morris SR, Mirza S. Medical students teaching medical students surgical skills: the benefits of peer-assisted learning. *J Surg Educ*. 2018;75(6):1471-1474. https://doi.org/10.1016/j.jsurg.2018.03.011
5. Abay EŞ, Turan S, Odabaşı O, Elçin M. Who is the preferred tutor in clinical skills training: physicians, nurses, or peers? *Teach Learn Med*. 2017;29(3):247-254. https://doi.org/10.1080/10401334.2016.1274262
6. Denadai R, Toledo AP, Oshiiwa M, Saad-Hossne R. Acquisition of suture skills during medical graduation by instructor-directed training: a randomized controlled study comparing senior medical students and faculty surgeons. *Updates Surg*. 2013;65(2): 131-140. https://doi.org/10.1007/s13304-013-0199-y
7. Ong MN, Lew KM, Cheong YJ, et al. Can we use peer-assisted learning to teach basic surgical skills? *Malays J Med Sci*. 2020; 27(5):101-107. https://doi.org/10.21315/mjms2020.27.5.10
8. Bulte C, Betts A, Garner K, Durning S. Student teaching: views of student near-peer teachers and learners. *Med Teach*. 2007;29(6): 583-590. https://doi.org/10.1080/01421590701583824
9. Thomas PA, Kern DE, Hughes MT, Tackett SA, Chen BY, eds. *Curriculum Development for Medical Education: A Six-Step Approach*. 4th ed. Johns Hopkins University Press; 2022.
10. Shumard KM, Denney JM, Quinn K, et al. Effectiveness of vaginal delivery simulation in novice trainees. *Fam Med*. 2016;48(9):696-702.
11. Holmström SW, Downes K, Mayer JC, Learman LA. Simulation training in an obstetric clerkship: a randomized controlled trial. *Obstet Gynecol*. 2011;118(3):649-654. https://doi.org/10.1097/AOG.0b013e31822ad988
12. Rees EL, Quinn PJ, Davies B, Fotheringham V. How does peer teaching compare to faculty teaching? A systematic review and meta-analysis. *Med Teach*. 2016;38(8):829-837. https://doi.org/10.3109/0142159X.2015.1112888
13. Rudolph JW, Raemer DB, Simon R. Establishing a safe container for learning in simulation: the role of the presimulation briefing. *Simul Healthc*. 2014;9(6):339-349. https://doi.org/10.1097/SIH.0000000000000047
14. Burgess A, van Diggele C, Roberts C, Mellis C. Feedback in the clinical setting. *BMC Med Educ*. 2020;20(suppl 2):460. https://doi.org/10.1186/s12909-020-02280-5
15. Pendleton D, Schofield T, Tate P, Havelock P. *The Consultation: An Approach to Learning and Teaching*. Oxford University Press; 1984.

Copyright © 2025 Bohrer and Rodgers. This is an open-access publication distributed under the terms of the Creative Commons Attribution license.

16. Klumpp S, Rocha I, Amirfazli C, et al. Impact of early procedural exposure on pre-clinical medical students' confidence. *Cureus*. 2025;17(1):e77411. https://doi.org/10.7759/cureus.77411

17. Procedural skills. In: Wald DA, Ander DS, Fisher J, et al. *Emergency Medicine Clerkship Primer: A Manual for Medical Students*. Clerkship Directors in Emergency Medicine; 2011:97-101.

18. Bilger N, Hinssen H, Rodgers DL. Using an inexpensive video endoscope to teach laryngoscopy (SimVentors Presentations). Poster presented at: International Meeting on Simulation in Healthcare; January 19-22, 2020; San Diego, CA.

19. Bevan J, Russell B, Marshall B. A new approach to OSCE preparation—PrOSCEs. *BMC Med Educ*. 2019;19(1):126. https://doi.org/10.1186/s12909-019-1571-5

20. Keating EM, O'Donnell EP, Starr SR. How we created a peer-designed specialty-specific selective for medical student career exploration. *Med Teach*. 2013;35(2):91-94. https://doi.org/10.3109/0142159X.2012.731110

21. Rees EL, Quinn PJ, Davies B, Fotheringham V. How does peer teaching compare to faculty teaching? A systematic review and meta-analysis. *Med Teach*. 2016;38(8):829-837. https://doi.org/10.3109/0142159X.2015.1112888

22. Lockspeiser TM, O'Sullivan P, Teherani A, Muller J. Understanding the experience of being taught by peers: the value of social and cognitive congruence. *Adv Health Sci Educ Theory Pract*. 2008;13(3):361-372. https://doi.org/10.1007/s10459-006-9049-8

23. Mezirow J. Transformative learning: theory to practice. *New Dir Adult Contin Educ*. 1997;1997(74):5-12. https://doi.org/10.1002/ace.7401

24. Clanton J, Gardner A, Cheung M, Mellert L, Evancho-Chapman M, George RL. The relationship between confidence and competence in the development of surgical skills. *J Surg Educ*. 2014;71(3):405-412. https://doi.org/10.1016/j.jsurg.2013.08.009


**Received:** February 13, 2025
**Accepted:** July 7, 2025
**Published:** August 19, 2025

Copyright © 2025 Bohrer and Rodgers. This is an open-access publication distributed under the terms of the Creative Commons Attribution license.
